# First person – Precious Obioha Owuamalam

**DOI:** 10.1242/bio.062616

**Published:** 2026-04-27

## Abstract

First Person is a series of interviews with the first authors of a selection of papers published in Biology Open, helping researchers promote themselves alongside their papers. Precious Obioha Owuamalam is first author on ‘ Nonsense mutations can increase mRNA levels’, published in BiO. Precious Obioha conducted the research described in this article while a The Darwin Trust of Edinburgh PhD research fellow in Dr Saverio Brogna's lab at University of Birmingham, Birmingham, UK. He is now a postdoctoral researcher in the lab of Dr Guilherme Costa at Queen's University Belfast, Belfast, UK, investigating how the spatial control of RNA helps regulate cell behaviour in health and disease.

**Figure:**
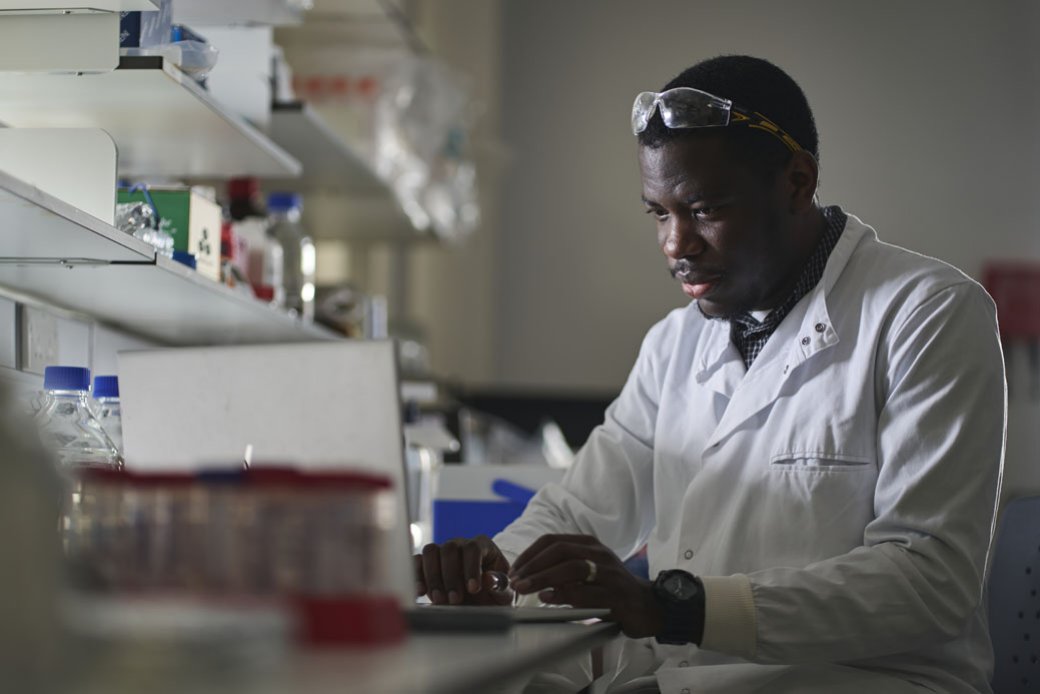
Precious Obioha Owuamalam

**Describe your scientific journey and your current research focus**

I studied microbiology at Michael Okpara University of Agriculture, Umudike (MOUAU), Nigeria, where my undergraduate research examined the microbial quality of commercially sold ice cream in Umuahia. That project showed that locally produced ice cream often exceeded acceptable microbial safety limits, highlighting under-recognised public health risks, especially for the vulnerable population, and sustaining my long-term interest in research with real-world impact.

During my degree, I also completed a 6-month internship in the Quality Assurance Department of the Nigerian Bottling Company in Owerri, where I gained hands-on experience in applied laboratory practice and quality control.

After graduating, I went on to gain more practical laboratory experience by working as an assistant laboratory technologist at a medical research laboratory, and afterward completed Nigeria's National Youth Service Corps programme, during which I taught biology to senior secondary school students for 1 year and also volunteered as an end-process independent monitor on polio supplementary immunisation activity with the World Health Organization.

I later received a Young African Leaders Initiative (YALI) Fellowship in Public Policy and Management, which broadened my perspective on how science, health and policy intersect. While building these experiences, I was applying for postgraduate scholarship/fellowship opportunities and was eventually awarded a The Darwin Trust of Edinburgh scholarship to undertake doctoral research in RNA biology at the University of Birmingham. My PhD focused on understanding how pre-mRNA splicing is functionally coupled to nonsense-mediated mRNA decay in the fission yeast *Schizosaccharomyces pombe*. That work deepened my interest in fundamental questions of gene regulation and how RNA processing shapes cell function. For my postdoctoral research, I shifted focus to RNA localisation and vascular biology at Queen's University Belfast. My current work investigates how cytoskeletal remodelling proteins coordinate mRNA localisation in endothelial cells during angiogenesis and endothelial barrier remodelling.

**Who or what inspired you to become a scientist?**

My path into science was shaped by a mixture of early ambition, curiosity and the people around me. In primary school, I used to say I wanted to become a professor, although at the time I understood it simply as someone who embodied knowledge and expertise. My classmates, teachers, friends and even family friends often called me ‘Prof.’, which, in a way, made that identity feel real long before I fully understood what it meant. At different stages, I was drawn to different subjects, and in secondary school I became especially interested in geography and even imagined becoming a geoscientist. That interest was deepened by a book my parents bought me, ‘*My Story of Oil and Gas*’, although I also loved chemistry and biology. When I applied to university, I applied for both geology and microbiology, but I eventually went on to study microbiology.

A major influence during my university years was reading about the lives of scientists, especially the short biographies in the ‘*Fundamentals of Analytical Chemistry*’ by Skoog, West, Holler and Crouch, which I always looked forward to. I have remained a huge fan of scientists' biographies because they make discovery feel deeply human and show how persistence, curiosity and imagination can shape a career. I was also inspired by Nigerian academics and scientists whose work I admired, especially those who trained abroad and returned to build distinguished careers, while my parents of blessed memory, both educators, gave me the encouragement and freedom to pursue my interests seriously.

My sister, Lynn, was another important influence, as she introduced me to yeast long before I knew it would later become the model organism for my PhD. So, looking back, my inspiration came not from one single moment, but from a gradual build-up of curiosity, role models and support that made science feel like home.

**How would you explain the main finding of your paper?**

mRNA molecules are the vital link between genes and proteins, carrying genetic instructions from DNA to the cellular machinery that makes proteins. In our study, we looked at what happens when the mRNA contains an early ‘stop’ signal, which tells the cell to stop making a protein too soon. Scientists often expect this kind of signal to reduce the amount of that mRNA because cells usually detect faulty mRNAs and break them down. We tested this by placing early stop signals at different points in several mRNAs in yeast cells, and we found that when the stop signal appeared near the beginning, the amount of the mRNA usually went down, but when it appeared further along, the effect was often small, and in some cases the amount of the mRNA actually increased. This was surprising because it shows that these genetic changes do not always cause mRNAs to be destroyed. In some situations, they can even make the mRNA more abundant. We also showed that the exact position of the stop signal, and how the mRNA is processed inside the cell, can strongly affect the outcome. Overall, our results show that an early stop signal does not always reduce the amount of mRNA; in some cases, it can unexpectedly increase it. This means the effects of these kinds of genetic changes may be more varied than scientists once thought. These findings could help researchers better understand genetic diseases and improve the design of genes used in biotechnology and research.

… nonsense mutations are not simply ‘message-destroying’ events

**What are the potential implications of this finding for your field of research?**

Our findings suggest that the effects of nonsense mutations are much more context dependent than the field has often assumed. Rather than always reducing mRNA levels, these mutations can leave them unchanged or even increase them, depending on factors such as where the stop signal occurs, whether the RNA is spliced and features at the end of the transcript. This means we need to think beyond RNA decay alone and consider a broader regulatory context in which transcription, RNA processing, translation and decay are tightly interconnected. It also has important implications for interpreting disease-causing mutations, because some nonsense variants may still allow relatively high RNA levels or even produce shortened but functional proteins. More broadly, the work reinforces the idea that RNA fate is shaped by multiple linked processes rather than by a single surveillance pathway. It also has practical value for designing reporter systems, synthetic constructs and other gene-expression tools. Features such as stop-codon position, splicing status and the distance to the end of the mRNA may all need to be considered carefully when designing expression systems. Overall, I think the main implication is that nonsense mutations are not simply ‘message-destroying’ events. They can reveal a much more nuanced and interconnected view of gene regulation than current models often allow.

**Figure:**
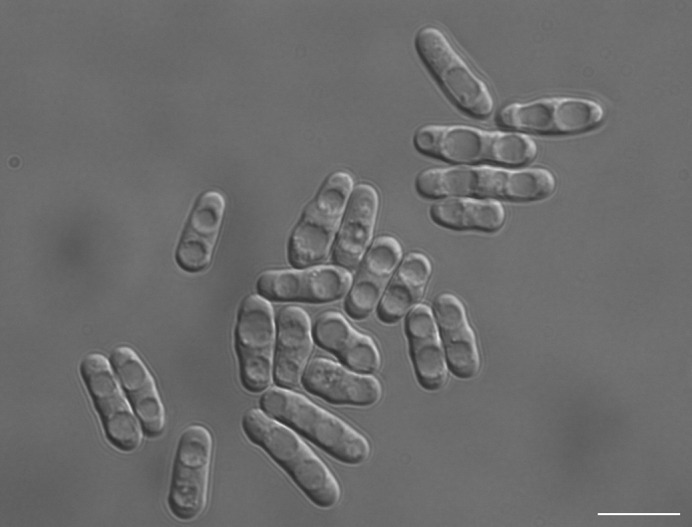
**A differential interference contrast image of *Schizosaccharomyces pombe* cells in response to transcriptional arrest induced by 1,10-phenanthroline.** Scale bar: 10 µm.

**Which part of this research project was the most rewarding?**

The most rewarding part was both the discovery itself and the process of getting there. One moment I remember very clearly was during a long late-night imaging session, when I noticed that one of the nonsense mutations produced a much brighter GFP signal than the wild-type control. My first thought was that I must have mixed up the cultures, so that same night I prepared the DNA for sequencing to confirm the strain. While waiting for the sequencing results, I repeated the imaging the next day with fresh replicates and saw the same pattern again. When the sequencing confirmed that the mutation was exactly what I thought it was, it was a really exciting moment because it pointed to something unexpected and biologically meaningful.

Another very rewarding aspect was the methodological side of the project. To generate the nonsense mutations, I initially tried several commercial kits and published protocols, but none worked well for what I needed, and the kits were also quite costly. In the end, I developed a simple home-made mutagenesis approach that proved both fast and efficient. That was especially satisfying because it not only enabled the project to move forward, but also produced a practical and affordable tool with value beyond this specific study. Overall, the most rewarding part was seeing persistence turn both technical setbacks and unexpected results into genuine discovery.

… it is often through things not working that you learn the most, sharpen your thinking and move forward more effectively

**What do you enjoy most about being an early-career researcher?**

What I enjoy most about being an early-career researcher is the combination of opportunity, growth and discovery. At this stage, there is still so much room to explore new ideas, build collaborations and shape your own scientific identity, and I have been very fortunate to benefit from a strong support network over the years. I also really value the day-to-day experience of research itself: being able to go into the lab, design experiments, test hypotheses and gradually build evidence towards a conclusion. There is something deeply satisfying about moving from a question to a result, especially when the outcome is unexpected and pushes your thinking in a new direction. For me, that mix of curiosity, possibility and hands-on discovery is one of the most rewarding parts of being an early-career researcher.

**What piece of advice would you give to the next generation of researchers?**

One piece of advice I would give is not to be afraid of failure. Failure is an essential part of science, and it is often through things not working that you learn the most, sharpen your thinking and move forward more effectively. I think it is especially important to fail early, learn from it and not let it stop you from taking opportunities. Be willing to apply, to ask questions, to try new approaches and to step into spaces that may feel intimidating.

I would also strongly encourage early-career researchers to build networks and seek mentors beyond their immediate environment. I have been very fortunate to benefit from generous mentors throughout my career, including Professor David Tollervey, whom I met through the RNA Society mentoring programme in 2020, and Dr Elizabeth Ballou, who was my first PhD internal examiner. Neither was my direct supervisor, but both have continued to offer valuable support, guidance, encouragement and perspective at important stages of my career, including times when I felt close to giving up.

Finally, I think it is very important to give back. Supporting students and fellow early-career researchers to realise their potential and progress to ambitious next steps has been one of the most rewarding parts of my academic career. To me, science is strongest when we not only pursue discovery, but also actively help others along the way.

**What's next for you?**

I am currently in a postdoctoral position and am now exploring opportunities to move towards independence. My goal is to secure an independent role that will allow me to establish my own research identity, build a competitive research programme in RNA biology and, eventually, develop my own research group. Scientifically, I am particularly interested in how the spatial control of RNA regulates cell behaviour in health and disease. Just as importantly, I want to build a collaborative and inclusive research culture in which trainees are supported to become confident, independent scientists, and where scientific excellence is matched by strong support for wellbeing, mentorship and lasting societal impact.
